# Real-time classification of tumour and non-tumour tissue in colorectal cancer using diffuse reflectance spectroscopy and neural networks to aid margin assessment

**DOI:** 10.1097/JS9.0000000000001102

**Published:** 2024-01-22

**Authors:** Scarlet Nazarian, Ioannis Gkouzionis, Jamie Murphy, Ara Darzi, Nisha Patel, Christopher J. Peters, Daniel S. Elson

**Affiliations:** a Department of Surgery and Cancer; b Hamlyn Centre for Robotics Surgery, Imperial College London, London, UK

**Keywords:** cancer, colorectal, diffuse reflectance spectroscopy, machine learning, neural network, surgery

## Abstract

**Background:**

Colorectal cancer is the third most commonly diagnosed malignancy and the second leading cause of mortality worldwide. A positive resection margin following surgery for colorectal cancer is linked with higher rates of local recurrence and poorer survival. The authors investigated diffuse reflectance spectroscopy (DRS) to distinguish tumour and non-tumour tissue in ex-vivo colorectal specimens, to aid margin assessment and provide augmented visual maps to the surgeon in real-time.

**Methods:**

Patients undergoing elective colorectal cancer resection surgery at a London-based hospital were prospectively recruited. A hand-held DRS probe was used on the surface of freshly resected ex-vivo colorectal tissue. Spectral data were acquired for tumour and non-tumour tissue. Binary classification was achieved using conventional machine learning classifiers and a convolutional neural network (CNN), which were evaluated in terms of sensitivity, specificity, accuracy and the area under the curve.

**Results:**

A total of 7692 mean spectra were obtained for tumour and non-tumour colorectal tissue. The CNN-based classifier was the best performing machine learning algorithm, when compared to contrastive approaches, for differentiating tumour and non-tumour colorectal tissue, with an overall diagnostic accuracy of 90.8% and area under the curve of 96.8%. Live on-screen classification of tissue type was achieved using a graduated colourmap.

**Conclusion:**

A high diagnostic accuracy for a DRS probe and tracking system to differentiate ex-vivo tumour and non-tumour colorectal tissue in real-time with on-screen visual feedback was highlighted by this study. Further in-vivo studies are needed to ensure integration into a surgical workflow.

## Introduction

Highlights

- Colorectal cancer is the third most commonly diagnosed and second leading cause of mortality worldwide.
- Positive circumferential and longitudinal resection margins are associated with higher rates of recurrence and worse survival.
- Diffuse reflectance spectroscopy (DRS) is an optical technique that is able to discriminate tissue type.
- Patients undergoing elective colorectal surgery were recruited and a hand-held DRS probe was used on the surface of the colorectal specimens after resection.
- Spectral data was acquired for normal and tumour tissue, and binary classification applied.
- A total of 3632 mean spectra for normal tissue and 4330 mean spectra for tumour tissue was obtained from 23 patients.
- The convolutional neural network -based SpectNet classifier was able to differentiate normal from tumour colorectal tissue with an accuracy of 91%, sensitivity of 93% and specificity of 88%. the area under the curve of the SpecNet classifier was 97%.
- Real-time tissue classification was presented on a user interface using a graduated colourmap.
- We have successfully shown a high diagnostic accuracy for a DRS probe and tracking system to differentiate tumour and non-tumour colorectal tissue in real-time with on-screen visual feedback.

Colorectal cancer (CRC) is the third most commonly diagnosed malignancy and the second leading cause of mortality worldwide^1^. Given the ever-ageing population, the global burden of this disease is projected to continue to increase in future decades^2^. Surgery is the mainstay of treatment for localised disease in CRC, and the quality of the procedure can have significant influence over outcome^3^. Surgery aims to minimise local recurrence and prolong disease-free survival (cancer-specific) by completely resecting tumour whilst sparing as much healthy tissue as possible. To ensure optimal results, it is crucial that there is no tumour invasion of the resection margin. In other words, one must achieve a negative circumferential resection margin (CRM) and longitudinal resection margin (LRM)^4^. Studies have shown that a positive CRM is associated with higher rates of recurrence and worse survival in CRC^4,5^. Similarly, a positive LRM has been associated with local recurrence, development of distance metastasis and lower disease-free survival^6–9^. Despite the best efforts to achieve a negative resection margin, detection of microscopically positive resection margins remain evident on postoperative histopathological assessment^10,11^. Thus, accurate intraoperative detection of resection margins and seamless integration into the surgical workflow are crucial for colon cancer care.

Diffuse reflectance spectroscopy (DRS) is an optical technique that allows the discrimination of tissue type based on the biochemical composition and structure of individual tissue. It is based on the amount of light that is scattered by structures within tissue and absorbed back onto the DRS probe. In contrast to advanced micro-endoscopic probes, DRS offers advantages in terms of affordability and simplicity, as it eliminates the need for contrast agents and invasive techniques. This technique has been used widely in recent years to aid early cancer detection. Studies have shown promising results for breast, lung, head and neck, oesophageal and stomach cancers^12–15^. In addition, there has been significant interest in the use of DRS in CRC^16–22^. These studies have been able to discriminate tumour from normal colon tissue with high overall accuracy of 0.95^16^, 0.94^17^ and 0.91^18^. However, the success thus far in the use of DRS technology for assessing colorectal specimens has lacked approaches which would make it clinically relevant and integrate smoothly into the operating field. For example, use of the DRS probe on the tissue was point-based, only a small number of spectra was acquired per sample point on the tissue and there was a disruption of the serosal layer in some studies, which hinders CRM measurement.

Despite its challenges which have hindered the widespread clinical adoption of DRS, ongoing research focusses on optimising DRS systems, improving data analysis algorithms, and addressing these challenges to enhance its clinical utility for CRC detection. In this study, we present an end-to-end DRS-based system that allows real-time colorectal tissue discrimination and DRS probe detection and tracking^23^, which is able to overcome the point-based limitation of the DRS probe and provide direct visualisation of the sampled areas on the specimen to the operator in real-time. Therefore, the primary aim of this study was to use DRS to distinguish cancer and non-cancer colorectal tissue in ex-vivo colorectal specimens, and produce augmented visual maps of the classification system in real-time.

## Methods

### Study design

This was a prospective validation study. Ethical approval was sought from the Harrow Research Ethics Committee (ref. no. 08/H0719/37). Patients undergoing elective colorectal cancer resection surgery at Imperial College NHS Trust in London were prospectively recruited between April 2021 and July 2022. Abdominoperineal excision of rectum (APER) cases were excluded from this study, since we only collected colonic tissue data. This was due to the presence of mesorectal fat surrounding the rectum. Normal tissue data were included for analysis for cases confirmed as T0 on formal histology.

### DRS set-up and probe tracking

**Figure 1:**
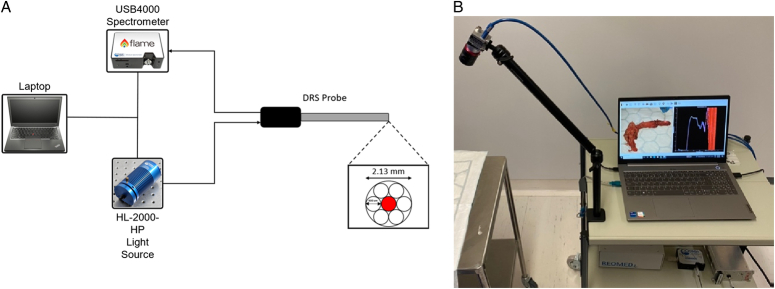
Diffuse reflectance spectroscopy (DRS) instrumentation for ex-vivo data acquisition: (A) schematic diagram of set-up; (B) photograph of set-up. The DRS probe was connected to both the light and the spectrometer to allow for data acquisition and sample illumination. All electronic devices communicated with proprietary software designed with Python version 3.6 (Python Software Foundation) on the laptop^23^.

The DRS equipment set-up and real-time probe tracking using a green-coloured marker have been described previously^23^. The set-up consisted of a hand-held fibre probe (Ocean Optics, QR600-7-SR-125F, Largo, USA), a tungsten halogen light source (Ocean Optics Inc., HL-2000-HP), a spectrometer (Ocean Optics Inc., USB4000), a camera (C920, Logitech International S.A., Lausanne, Switzerland) and a laptop computer. The probe contained six peripheral illumination fibres together with a central light collection fibre in a cylindrical configuration. A schematic representation and photograph of the instrumentation for data acquisition is shown in Figure 1.

Probe tracking was achieved using a two-dimensional (2D) colour-based optical marker tracking system. A green colour marker was attached to the distal end of the DRS fibre probe and thus, localisation of the probe tip was enabled. HSV segmentation was used for the extraction of the 2D position of the probe tip, while a standard Kalman filter was employed for tracking its location. Probe tracking was performed in real-time at 30 frames-per-second^23^.

### Data collection and processing

Freshly resected colorectal specimens were sampled within 15 min of termination of the blood supply using the hand-held DRS probe inside the operating theatre. Spectral data were obtained by probing the outer surface of the specimen over a non-tumour, or normal, tissue area and over the suspected tumour area. These areas were identified by visual and haptic inspection by a surgeon. Approximately 200 spectra were acquired for each tissue type per specimen. Normal tissue was chosen as far away from the suspected tumour area as possible, and based on an area with minimal overlying fat tissue, and no staples or sutures.

Software was developed to include all the functionalities of our system, namely spectral data acquisition and processing, spectral classification, and probe tracking. A user-friendly graphical user interface allowed the user to directly visualise optical biopsy sites, with the aid of the probe tracking system, in real-time.

**Figure 2:**
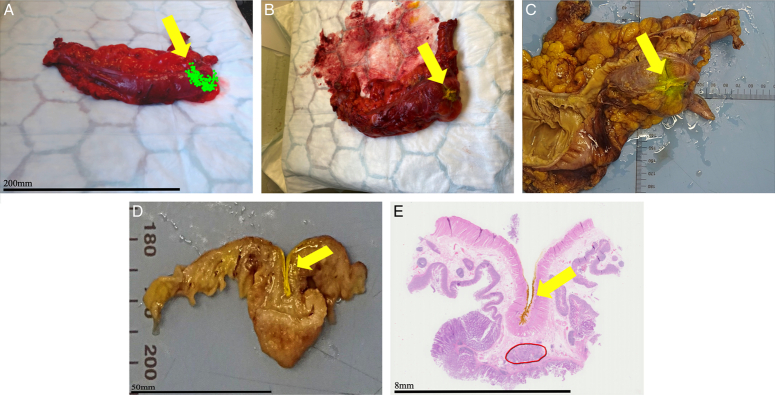
Histopathology correlation. (A) Photo of scanned colon tissue with tracked diffuse reflectance spectroscopy locations over suspected tumour area indicated. (B) Photo of colon tissue with painted yellow dye over the suspected tumour tissue. (C) Photo of colon tissue after being in formalin with yellow tissue dye visible. (D) Photo of sliced colon tissue with yellow dye visible over the suspected tumour. (E) Scanned hematoxylin and eosin slide showing yellow tissue dye over confirmed tumour tissue (marked in red).

Immediately after data collection (10 min), the area sampled as tumour tissue was marked with yellow tissue dye (Cancer Diagnostics Inc.). The specimen was then sent to the histopathology department. Photos were taken by the dissector of the sliced tissue, including areas containing yellow dye^15^. Standard protocols for processing the specimen were followed. Areas marked with yellow dye which were confirmed by the pathologist as tumour, were labelled as ground truth and used to train the machine learning classifiers. The histopathology correlation is shown in Figure 2.

### Classification methodology

Upon data collection, spectral normalisation and noise reduction were performed, using a white reflectance standard and dark field readings to account for inter-patient, background light, and signal quality variability. After that, the spectral data were normalised by removing the mean and scaling to unit variance, using the standard normal variate (SNV) method^24^. An automated method was implemented for outlier (e.g. erroneous measurements or air interference) detection and removal. For this method, the median of the residuals was calculated, along with the 25^th^ and 75^th^ percentiles. The difference between each historical value and the residual mean was then calculated. If the historical value was 1.5 times the median absolute deviation (MAD) away from the median of the residuals, that value was classified as an outlier. In addition to the intensity measurements, spectral shape-based features were extracted and used for classification, comprising the intensity value and wavelength of the greatest peak for each spectrum, the intensity and wavelength of the secondary peak, and the mean intensity in three spectral regions, namely 480–550 nm, 580–700 nm, and 450–720 nm. The haemoglobin saturation coefficient was also derived, using Monte Carlo simulations, to quantify the effect of changing haemoglobin saturation on the curve features. To reduce overfitting and improve discrimination accuracy, we performed the Boruta feature selection method on the data^25^. A stratified 5-fold cross-validation was then performed using tree-based classifiers like random forest (RF) and and Extreme Gradient Boosting and Light Gradient Boosting Machine (XGB, LGBM) as they are effective with highly complex data^26–28^. Conventional machine learning algorithms were also used to differentiate tumour and non-tumour tissue, such as linear support vector machine (SVM), and a shallow neural network, Multi-Layer Perceptron (MLP). In addition, a one-dimensional convolutional neural network (CNN), named SpecNet, was developed and used. These classifiers were finally evaluated in terms of sensitivity, specificity, overall accuracy, and the area under the receiver-operator characteristic (ROC) curve.

**Figure 3:**
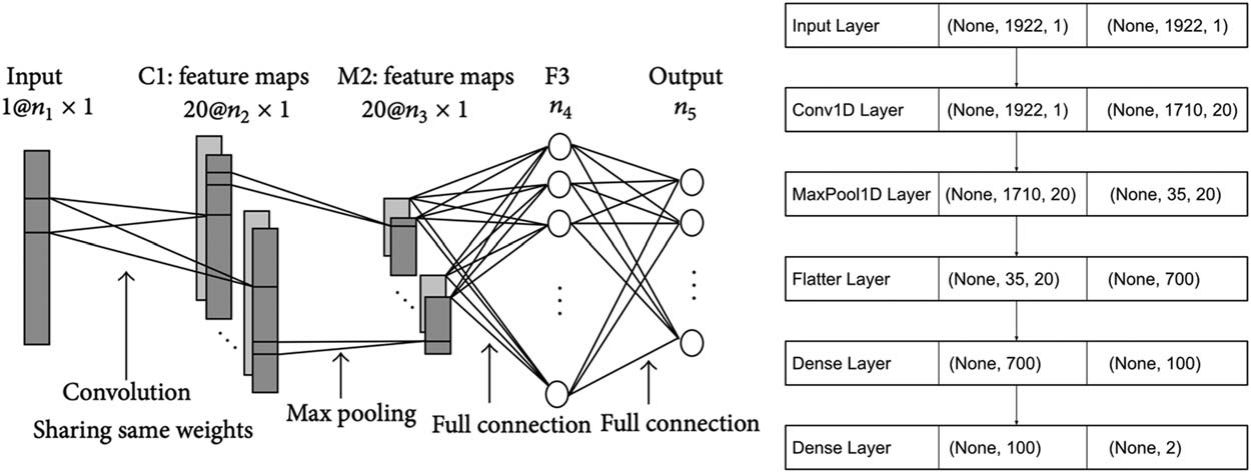
The architecture of the proposed one-dimensional convolutional neural network for colorectal tissue classification. The SpecNet is a five-layer convolutional neural network for learning visual representations in diffuse reflectance spectroscopy data. It uses 20 kernels to filter the input data, and the output layer classifies spectra based on the spectral band size and number of output classes in the dataset.

The hierarchical architecture of CNNs is proving to be the most efficient and successful way to learn visual representations. Figure 3 shows the hierarchical architecture of the proposed CNN-based SpecNet for spectral classification. The network contains five layers with weights, including the input layer, convolutional layer C1, max pooling layer M2, fully connected layer F3, and output layer. Each spectral measurement in DRS can be regarded as a 2D image, and the size of the SpecNet input layer is (n1, 1), where n1 is the number of spectral bands (1922 in this study). This treatment acknowledges the inherent multidimensionality of the data, condensing the spectral information across the bands into a single-channel representation akin to an image with a single height dimension. The first hidden convolutional layer C1 filters the input data with 20 kernels of size k1×1. Layer C1 contains 20×n2×1 nodes, and there are 20×(k1 + 1) trainable parameters between layers C1 and the input layer. The second hidden layer is the max pooling layer M2 with a kernel size of (k2, 1) and contains 20×n3×1 nodes, where n3 =n2 / k2. The fully connected layer F3 has n4 nodes, and there are (20×n3 + 1)×n4 trainable parameters between this layer and layer M2. The output layer has n5 nodes, and there are (n4 + 1)×n5 trainable parameters between this layer and the F3 layer. The output data of the neural network comprises binary class predictions that decisively assign each input spectrum sample to either of the two classes: healthy tissue or cancerous tissue. These binary class predictions serve as a categorical affirmation of the network’s evaluation of each individual pixel’s spectral attributes. Upon traversing the network’s layers, which include convolutional, pooling, and fully connected components, the model collectively aggregates and processes spectral patterns representative of each tissue type. This culminates in the final layer, where the network furnishes a definitive declaration regarding the classification label for each pixel—either “healthy” or “tumour.”

Python 3.6 (Python Software Foundation) was used for feature engineering and machine learning classification, and MATLAB R2020b (MathWorks) was used for data processing. For the training and validation of the SpecNet, TensorFlow deep learning framework was employed.

### Statistical analysis

To ensure unbiased and reliable performance evaluation, the dataset was methodically divided into distinct training and testing sets, adhering to a patient-wise grouping framework. This strategy ensured that data originating from each patient was collectively allocated to either the training or testing set, circumventing any inadvertent intermixing that might lead to data leakage or skewed assessments. The rationale underpinning this patient-wise grouping strategy is to reflect the patient-specific variability encountered in real-world clinical scenarios.

A stratified five-fold cross-validation methodology was employed exclusively on the training sub-set. This five-fold approach entailed the subdivision of the training data into five equitably balanced subsets, characterised by an equitable representation of both tumorous and healthy tissue samples representing the real-world distribution. Within this stratified five-fold cross-validation framework, the training process was iteratively executed four times, with one sub-set reserved for validation, effectively mitigating the potential for overfitting and ensuring a comprehensive model assessment.

The machine learning classifiers were evaluated in terms of sensitivity, specificity, overall accuracy, and the area under the curve (AUC). Overall accuracy was calculated as the proportion of correctly identified spectra over a total number of spectra. ROC curves were plotted.

## Results

### Patient characteristics

A total of 23 patients were recruited into the study. The majority of patients undergoing colorectal cancer surgery were male (74%). The age of patients ranged from 50 to 88 years old, with a median age of 70. Most patients (*n*=14; 61%) underwent an anterior resection procedure, whilst 8 (35%) patients underwent a right hemicolectomy and only 1 (4%) patient underwent a sigmoid colectomy. There were three patients with T0 disease on histology. These were found to be benign adenomatous polypoid lesions. Normal tissue data were collected for these patients. Table 1 outlines the patient characteristics.

**Table 1:** Patient cohort characteristics.

Characteristic	*N* (%)
Age, median (range)	70 (50–88)
Sex	
Female	6 (26.0)
Male	17 (74.0)
Procedure	
Anterior resection	14 (61.0)
Right hemicolectomy	8 (35.0)
Sigmoid colectomy	1 (4.0)
Tumour histology	
Adenocarcinoma	20 (87.0)
Benign adenomatous polyp	3 (13.0)
Tumour stage	
0	3 (13.0)
1	5 (21.7)
2	2 (8.7)
3	11 (47.8)
4	2 (8.7)
Neoadjuvant therapy	
Chemotherapy	1 (4.3)
Chemoradiotherapy	0
Radiotherapy	0
None	22 (95.7)

### Spectral analysis

**Figure 4:**
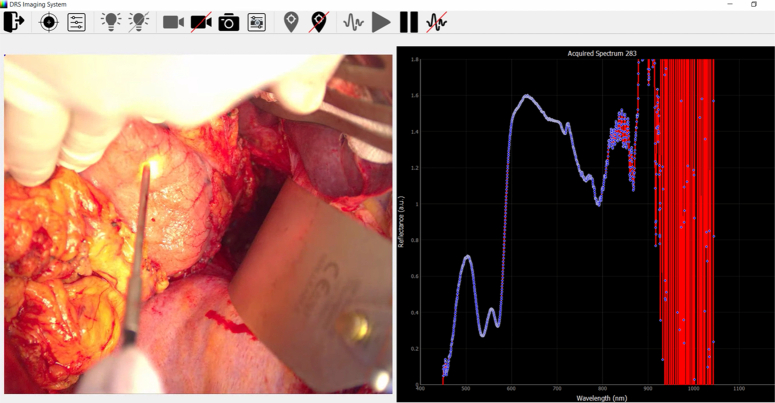
Python software for hardware system control, data acquisition and real-time classification. On the top, the control panel allows for execution of data acquisition protocol, probe tracking, hardware calibration and light source control. On the left panel, the live video feed from the RGB camera is displayed, while on the right panel the acquired reflectance data (420–1000 nm spectral range) for every optical biopsy tissue point is displayed in real-time.

**Figure 5:**
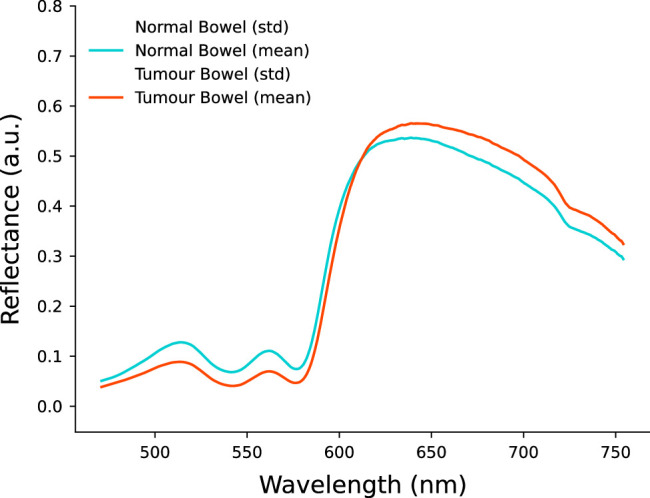
Mean spectra for colorectal tissue.

A total of 7692 mean spectra were included in this study. This consisted of 3632 mean spectra for non-tumour or normal tissue and 4,330 mean spectra for tumour tissue. Each processed mean spectrum contained 1922 equally spaced intensity measurements in the 450–1000 nm spectral range. The software interface of the DRS system is shown in Figure 4 and the means of all spectra for each of the tissue classes are shown in Figure 5.

### Classification results

**Figure 6:**
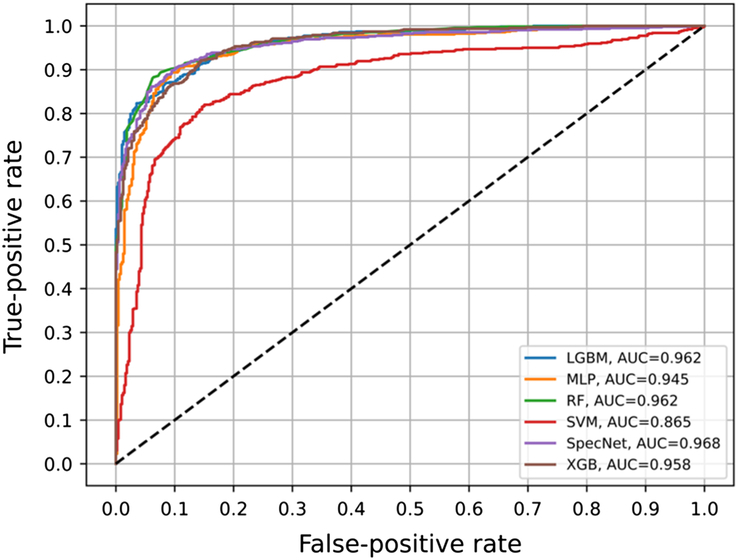
Receiver-operator characteristic curves for colorectal tissue. AUC, area under the curve; RF, random forest; SVM, support vector machine.

Results of the classification for colorectal spectral data are presented in Table 2. The 1D CNN and the tree-based classifiers (RF, XGB, LGBM) outperformed SVM. There was no significant difference when comparing the SpecNet and tree-based classifiers; however, overall, the SpectNet classifier performed better in terms of accuracy (0.91), sensitivity (0.93) and specificity (0.88). The AUC of the SpecNet classifier was 0.97. The ROC curves are shown in Figure 6. A possible reason for this finding may be due to the relatively small amount of spectral data used for training the SpecNet classifier, whereas deep neural networks require large amounts of data to show their relevance.

**Table 2:** Performance metrics for the spectral data classification using supervised machine learning and permutation feature importance for the feature selection.

	Colorectal tissue			
Classifier	Accuracy	Sensitivity	Specificity	AUC
SpecNet	90.8 (88.7–92.9)	93.0 (90.7–95.3)	88.0 (84.1–91.9)	96.8 (95.5–98.1)
RF	89.8 (87.5–92.1)	90.0 (87.9–92.1)	89.6 (86.4–92.8)	96.2 (94.9–97.5)
LGBM	89.3 (87.5–91.1)	89.9 (87.9–91.9)	88.6 (85.6–91.6)	96.2 (95.1–97.3)
XGB	88.7 (87.0–90.4)	89.2 (87.2–91.2)	88.2 (85.2–91.2)	95.8 (94.7–96.9)
MLP	88.8 (86.2–91.4)	89.9 (85.6–94.2)	87.4 (82.8–92.0)	94.5 (92.5–96.5)
SVM	80.5 (75.9–85.1)	80.5 (72.5–88.5)	80.5 (72.3–88.7)	86.5 (83.3–89.7)

Data showing the ability of multiple classifiers to detect normal versus cancerous tissue presented as mean (95% CI). Overall accuracy is calculated as the proportion of correctly identified spectra over the total number of spectra.

AUC, area under the curve; LGBM, Light Gradient Boosting Machine; MLP, Multi-Layer Perceptron; RF, random forest; SVM, support vector machine; XGB, Extreme Gradient Boosting.

It can be seen in Table 2 that the specificity of the RF classifier was better than SpecNet. The reasons for this observation may be due to a combination of factors, including, feature extraction and dimensionality, given that SpecNet is a 1D CNN whilst RF employs an ensemble learning approach that inherently accommodates the complex relationships and interactions within the dataset, and data availability and sample size, since RF can robustly handle scenarios with limited data points, making it well-suited for cases where the sample size is relatively small.

**Figure 7:**
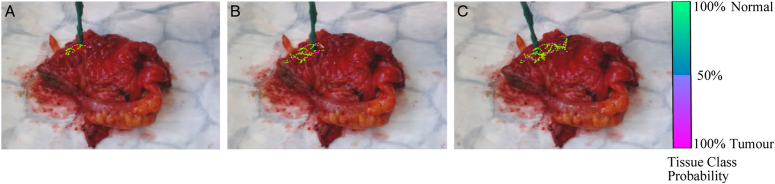
Real-time tracking at each optical biopsy site (A-C) coupled with the binary classification probability of each site (A-C) on a colorectal specimen.

Real-time tissue classification was achieved and presented on the user interface when using the DRS probe. Real-time tracking at each optical biopsy site coupled with the binary classification probability of each site was visualised as either normal (100% green) or tumour tissue (100% pink) using a graduated colourmap. Tissue type was highlighted on the screen in real-time during sampling, as illustrated in Figure 7.

Our study analysed 4330 mean spectra for tumour tissue, increasing the accuracy of results. The classification in this study was run on a sub-set of 13 patients (3117 mean spectra of tumour tissue), and then on the remaining 10 patients, resulting in a classification error of ±0.05. This ensured the statistical reproducibility of the proposed machine learning models upon following the aforementioned data transformations and model training hyperparameters.

## Discussion

In this study, we showed that a DRS probe and tracking system can be used to differentiate tumour and non-tumour colorectal tissue with a high diagnostic accuracy. The novel CNN-based SpecNet classifier, proposed in this study, was able to differentiate colorectal tumour and non-tumour tissue with a diagnostic accuracy of 0.91. A graphical user interface was able to project the tracked area on the tissue and classify tissue type in real-time to aid resection margin assessment.

This study has been able to show results that set it apart from previous studies exploring the use of DRS for colorectal cancer detection^16,18,19,29,30^. Dhar *et al.* ^30^. showed an accuracy of 80% for differentiation of colonic cancer from normal tissue, which was based on 31 spectra from colorectal cancer sites. Baltussen *et al.* ^16^. achieved a mean accuracy of 95% in differentiating colorectal tumour tissue from healthy bowel wall. However, the classifier used in their study was trained on 448 locations, with only 164 locations for tumour tissue. Langhout *et al.* ^18^. achieved an accuracy of 91% by analysing 603 separate tissue measurements, with 276 on tumour tissue, and Schols *et al.* ^19^. included 60 spectra from colonic tumour tissue. The small sample sizes in these studies limits their reliability and may lead to overfitting.

The national guidance from the United Kingdom Royal College of Pathologists recommends that the bowel is left intact at the level of tumour to preserve the circumferential margin, the assessment of which would otherwise be compromised by disrupting the specimen^31,32^. Studies investigating the use of DRS for colorectal cancer tissue assessment have used a variety of techniques for histopathological correlation. In the study by Langhout *et al.* ^18^., biopsies were taken from macroscopically recognised tumour tissue and sent off for histopathological assessment, whilst the gold standard used by Baltussen *et al.* ^16^. involved samples of tissue being taken from the specimen and put into cassettes before being sent off to the histopathology department. These techniques would have disrupted the integrity of the specimens, deeming them inappropriate for CRM assessment, and are not a true representation of the surgical workflow during colorectal cancer procedures. In this study, we were able to use yellow tissue paint to mark the area of suspected tumour tissue following collection of spectral data and follow this through to the hematoxylin and eosin (H&E) slide confirming a tumour diagnosis. This technique was quick, taking only a few minutes, simple and meant that the circumferential margin of the tissue was preserved. The light emitted by the DRS probe was able to penetrate colonic tissue allowing the system to detect tumour tissue in deeper muscular layers away from the serosal surface on which it was used. This was highlighted by the systems’ success in differentiating normal and tumour tissue in T2 or T3 tumours. However, we plan on further assessing this in future studies through sub-set analyses.

In this prospective validation study, we were able to collect a total of 7692 mean spectra immediately after resection of colorectal specimens intraoperatively, minimising the disruption to perfusion of the specimens. Data collection was complete within 15 min of specimens having been resected. This was much quicker compared to some previous studies in which data had been collected within an average time of 60 min after surgical resection^16,33^. It also ensured that the results were mirroring the in-vivo setting as much as possible. In addition to these findings, we have been able to show the possibility of real-time tissue discrimination live on-screen in the operating theatre during colorectal cancer procedures. We have been able to achieve this using a developed DRS tracking system and a graduated colourmap for tissue classification^15,23^.

In addition to this combination of techniques, our study has been further strengthened by the use of neural networks, which achieved an accuracy of over 90%. Neural networks allow analysis of large, complex datasets and make clinical application of the process more effective due to the inherent nature of learnt response^34–37^. Neural networks have been used in combination with optical techniques for intraoperative detection of cancers^38,39^. Unlike our study, other studies exploring the use of neural networks for colorectal cancer detection have either analysed mucosal tissue^40^, or have evaluated neural networks in view of using datasets to determine an optimal classification strategy^29^.

There are limitations to this study that should be noted. Firstly, this was a single-centre study with a total of 23 patients. A multi-centre study would help to recruit more patients and allow widening of the study population. Secondly, the specimens were sampled immediately after resection without any cleaning techniques being applied, resulting in differing quantities of stool or blood in the bowel lumen. Although this may have caused some inconsistencies with the data, the process reflects what would be expected if the system was applied to the in-vivo environment. Furthermore, we did not collect data on fat tissue and chose to collect normal tissue data from areas of the bowel without overlying fat to ensure consistent spectra. However, when collecting data for tumour, fat tissue could not always be avoided. In future studies, we will collect data for fat tissue and aim for this not to affect results. Lastly, one case underwent chemotherapy in this study which may have affected the data due to the presence of fibrosis. We excluded samples which were confirmed after histopathological analysis as fibrosis tissue, and the sparsity of the data did not allow further analysis. However, we would benefit from analysing fibrosis tissue in future studies, especially since this type of tissue is difficult to differentiate intraoperatively by visual and haptic feedback by the surgeon when making decisions about tumour margin assessment.

DRS measurements were performed from the exterior surface of the specimen, specifically the serosa, without necessitating opening of the lumen. An inherent challenge in such an approach lies in the potential variability in light penetration due to the heterogeneous nature of the tissue surface and the diverse underlying tissue properties. The depth to which light penetrates and the variability of this depth in manipulated tissues is critical for accurate DRS measurements. Studies were conducted to determine the penetration depth of light in different tissue types, and the results indicated certain predictable patterns in DRS changes associated with various tissue conditions^33,41^. It is important to understand that the chemical and physical characteristics of the tissue above the tumour may not exhibit homogeneity across the entire specimen’s outer surface. This variability can stem from factors like surgical manipulation, bleeding, or innate tissue characteristics. Such heterogeneities can potentially mask or alter the DRS signatures of underlying tumour tissue, posing challenges for real-time surgical applications. We are actively exploring calibration techniques that adjust for these variabilities, ensuring consistent and reliable measurements.

We have highlighted that the use of a DRS probe and tracking system can be successfully adopted intraoperatively for real-time tumour margin assessment in colorectal cancer with the aim of potentially aiding surgeons in decision-making processes. Given the success in our findings, we intend on expanding the study to include rectal cancer resections, since a positive CRM in this cohort has been shown to be an independent, poor prognostic factor for local recurrence and survival^42–44^. We plan to use DRS for assessment of tissue infiltration of the peritoneum and other organs in future studies, to be able to assess for metastasis of tumour in real-time during cancer procedures.

We have also developed a sterilisable probe to allow in-vivo measurements. An in-vivo setting will allow application of the DRS probe directly onto the surface of colorectal tissue prior to tissue resection and cessation of blood supply. Intraoperatively, the DRS system will be able to facilitate real-time tissue classification with live on-screen categorisation of “tumour” or “normal” tissue. This approach aims to enhance surgical decision-making by providing instantaneous feedback to the surgeon during the critical stages of the procedure, particularly in guiding margin assessment. The system will be able to be used both during open and laparoscopic surgery. For the latter, the DRS probe will be inserted through a port site and be used to scan the tissue with the assistance of laparoscopic instruments. Clinical application of the technique could be targeted to scenarios in which a high risk of incomplete resection is anticipated. It is vital that DRS is applied with high accuracy, but we have shown that it is also important for the system to be incorporated with standard surgical protocols and easily integrate into the surgical workflow in a way that will best aid surgeons.

## Conclusion

We have successfully shown a high diagnostic accuracy for a DRS probe and tracking system to differentiate tumour and non-tumour colorectal tissue in real-time with on-screen visual feedback. The importance of integrating such a system into the surgical workflow has been highlighted. Adoption of advanced deep learning neural networks to achieve accurate probe tip detection and tracking, as well as for optimal classification technique, should be developed further in future studies. We intend to employ DRS to evaluate peritoneal and organ tissue infiltration in subsequent studies, enabling assessment of tumour metastasis intraoperatively.

## Ethical approval

Ethical approval was sought from the Harrow Research Ethics Committee (ref. no. 08/H0719/37).

## Consent

Written consent was obtained from patients taking part in the study. All data were de-identified.

## Sources of funding

This study is funded by the National Institute for Health Research Imperial Biomedical Research Centre and the Cancer Research UK Imperial Centre.

## Author contribution

S.N.: study concept, design, data collection, analysis, writing paper, administration. I.G.: study concept, design, data collection, statistics, data analysis, writing paper. J.M.: study concept, review and editing. A.D.: study concept, supervision. N.P.: study concept, supervision. C.J.P.: study concept, design, review and editing, supervision. D.S.E.: study concept, design, review and editing, supervision.

## Conflicts of interest disclosure

A.D. is the Chair of Flagship Pioneering UK Ltd and the Pre-emptive Medicine and Health Security Initiative.

## Research registration unique identifying number (UIN)

National clinical trials - NCT05830292.

## Guarantor

Professor Daniel S. Elson.

## Data availability

The datasets generated during and/or analysed during the current study are not publicly available but are available from the corresponding author on reasonable request.

## Provenance and peer review

Not invited.
